# Epigallocatechin-3-gallate and zinc provide anti-apoptotic protection against hypoxia/reoxygenation injury in H9c2 rat cardiac myoblast cells

**DOI:** 10.3892/mmr.2015.3603

**Published:** 2015-04-08

**Authors:** XING ZENG, XUERUI TAN

**Affiliations:** Department of Cardiology, The First Affiliated Hospital of Shantou University Medical College, Shantou, Guangdong 515041, P.R. China

**Keywords:** zinc, epigallocatechin-3-gallate, hypoxia/reoxygenation, apoptosis, phosphatidylinositol 3-kinase/Akt pathway

## Abstract

It has previously been demonstrated that phospha-tidylinositol-3-kinase (PI3K)/Akt and cleaved caspase-3 serve critical roles in the apoptosis of cardiac myocytes following ischemia/reperfusion injury. Epigallocatechin-3-gallate (EGCG), the predominant catechin component of green tea, has been reported to have potential cardioprotective effects in primary cultures of cardiac myocytes exposed to I/R injury, mediated through inhibition of signal transducers and activators of transcription-1 activity. In addition, it is also known that the biological behavior of EGCG may be influenced by metal ions, for example the hepatoprotective activity of EGCG has been reported to be enhanced by zinc. In the present study, the protective effects of EGCG with zinc were assessed on cultures of rat cardiac myoblasts exposed to hypoxia/reoxygenation (H/R) injury. H9c2 cells were subjected to 3-h hypoxia, followed by 1-h reperfusion. EGCG and/or zinc were perfused prior to induced hypoxic stress. It was demonstrated that when EGCG interacted with zinc, the anti-apoptotic activity was significantly enhanced. To the best of our knowledge, the current study was the first to demonstrate that EGCG + Zn^2+^ protects H9c2 cells against H/R injury through activation of the PI3K/Akt pathway, as determined by western blotting. Since EGCG + Zn^2+^ may, at least in part, protect cardiac myocytes against H/R-induced apoptotic cell death, the PI3K/Akt pathway of EGCG may be enhanced by its interactions with zinc during H/R injury. Furthermore, it was suggested that a similar procedure may be implemented in a clinical setting, in order to maximize PI3K/Akt activation levels in patients with acute coronary artery disease. EGCG and zinc may therefore represent effective agents for use in the prevention of I/R injury in clinical practice.

## Introduction

Primary percutaneous coronary intervention (PCI) is, at present, the most commonly used management strategy for acute myocardial infarction (AMI) with ST-segment elevations. However, a significant number of patients receiving PCI fail to achieve complete and sustained myocardial reperfusion and therefore remain at risk of developing large infarcts (1). The in-hospital mortality of patients with AMI undergoing PCI is between 4 and 7% (2). The poor outcome amongst these patients has been attributed to the effects of the restoration of blood flow to previously ischemic tissue (3). Increases in knowledge have revealed that the common pathophysiological scenario, myocardial ischemia reperfusion injury, including ischemia/reperfusion (I/R) and hypoxia/reoxygenation (H/R) forms of injury, results in depressed myocardial function and harmful morphological alterations, which may lead to heart failure (4). Additionally, it may clinically manifest with myocardial necrosis, cardiac arrhythmia, myocardial stunning and microvascular dysfunction (5). Previous studies have identified apoptosis as a significant mechanism underlying cell death during I/R injury in cultured cardiac myocytes (6,7), and that the inhibition of this apoptosis is able to prevent I/R injury (8).

Green tea, a beverage consumed worldwide, has been suggested to possess significant health-promoting effects, while polyphenol (−)-epigallocatechin-3-gallate (EGCG), the predominant catechin from tea, has been reported to exert a variety of beneficial cardiovascular effects by influencing the activity of receptor and signal transduction kinases (9). In addition, the consumption of green tea by humans has been hypothesized to be associated with a lower incidence of coronary artery disease (10). A previous study identified that *in vivo* treatment with EGCG reduced I/R injury by inhibiting the nuclear factor-κB and activator protein 1 pathways in rat hearts (11). In addition, Townsend *et al* (12) reported that EGCG reduced signal transducers and activators of transcription-1 phosphorylation and protected cardiac myocytes against I/R-induced apoptotic cell death in isolated rat hearts. Furthermore, administration of EGCG *in vitro* was observed to prevent apoptosis of cardiomyocytes by regulating pro-apoptotic and anti-apoptotic proteins, including B cell lymphoma-2 (Bcl-2) and Bcl-2-associated X protein, and by simultaneously regulating caspase-3 in isolated rat hearts (13). EGCG has been suggested to inhibit cardiac myocyte apoptosis by preventing telomere shortening and telomere repeat-binding factor 2 loss (14). Therefore, EGCG may function as an effective anti-apoptotic agent.

Several previous studies have focused on evaluating the interactions of catechin with metal ions. Catechin interacts with metal ions, particularly transitional metal ions, which lead to alterations in the bioactivity of catechin (15,16). Zinc has been reported to be an essential biometal, which has various pleiotropic roles in biological systems (17). In reference to cell survival, zinc has been demonstrated to exhibit anti-apoptotic effects, acting either as an inorganic ion or as a key cofactor of various organic molecules. Previous studies have suggested that the cardioprotective effects of zinc against I/R injury are dependent on its ability to activate phosphatidylinositol-3-kinase (PI3K)/Akt signaling (18,19). It has been demonstrated that the activation of PI3K/Akt results in the growth and survival of cardiac myocytes. In addition, zinc-induced ErbB2 protein expression provides a clue, indicating that ErbB2 may act upstream of Akt activation and be essential in preventing reperfusion injury (18). These results have significance with regard to the preventive effects of zinc on cardiomyocytes against H/R-induced apoptosis.

Preliminary investigations led to the development of the process of ischemic preconditioning, which is a cardioprotective method against the development of irreversible damage following I/R, and demonstrates that I/R injury may be attenuated (20). The prophylactic use of pharmacological agents mimicking the effects of ischemic preconditioning may represent an effective strategy for reducing the extent of myocardial damage resulting from I/R or H/R. The PI3K/Akt pathway has been reported to be essential for cardioprotection in response to ischemic preconditioning (21). In a previous study, the anti-apoptotic effects of EGCG were observed in cardiomyocytes (12); however, it was reported that EGCG attenuated the I/R-induced phosphorylation of Akt in human umbilical vein endothelial cells (22). Thus, the role of EGCG and zinc in preconditioning and toxicity in H9c2 cells and its underlying mechanism of action are beginning to be elucidated. In the present study, the effectiveness of EGCG with exogenous zinc at an optimized concentration in terms of biological activity at ameliorating H/R injury was investigated using H9c2 cells *in vitro*. The biochemical mechanism underlying the cardio-protective effects was investigated by examining alterations in the expression levels of phosphorylated (p)-Akt and cleaved caspase-3 activity in the H9c2 cell model of H/R, in addition to the effects of EGCG and zinc on cell viability following H/R injury.

## Materials and methods

### Chemicals

Purified EGCG (>98%), zinc chloride, Dulbecco’s modified Eagle’s medium (DMEM) and fetal bovine serum (FBS) were purchased from Sigma-Aldrich (St. Louis, MO, USA). Human H9c2 cells were purchased from the Shanghai Institute of Cell Biology, Chinese Academy of Sciences (Shanghai, China). Western blotting was performed using specific antibodies against p-Akt and cleaved caspase-3 (Santa Cruz Biotechnology, Inc., Santa Cruz, CA, USA). All other chemicals were of extra-pure or analytical grade unless otherwise specified.

### Treatment of H9c2 cells

H9c2 cells were maintained in DMEM supplemented with 10% FBS, 2% l-glutamine, 10% sodium bicarbonate, 10% sodium pyruvate, 5% Hepes, 1% penicillin/streptomycin and 1% gentamycin (Sangon Biotech Co., Ltd., Shanghai, China) in an incubator (37°C, 95% air + 5% CO_2_). The cells were cultured for 1–2 days under these normal conditions, until they reached 90% confluence prior to anoxic treatment. EGCG (0, 5, 10, 15 and 20 *µ*M) and Zn^2+^ (0, 5, 10, 15 and 20 *µ*M) were added to the myocytes 30 min prior to reoxygenation. Hypoxic stress was implemented by incubating cultured H9c2 cells in serum- and glucose-free DMEM. The culture dish was placed in an airtight incubator at 37°C under an atmosphere of 95% N_2_ and 5% CO_2_ for 3 h followed by reoxygenation for 1 h in normal (37°C, 95% air + 5% CO_2_) conditions with 10% FBS-DMEM. EGCG and Zn^2+^ at various concentrations were added 30 min prior to the reoxygenation. Myocytes not exposed to H/R served as normoxic controls. At the end of the H/R treatment, myocytes were examined for viability and apoptosis by Hoechst 33258 (Beyotime Institute of Biotechnology, Haimen, China) staining and western blot analysis.

### MTT assays

MTT assays were performed in order to determine the effects of EGCG and Zn^2+^ on the growth of H9c2 cells. Cells were plated in 96-well tissue culture plates and treated with varying doses of EGCG (0, 5, 10, 15 and 20 *µ*M) or Zn^2+^ (0, 5, 10, 15 and 20 *µ*M) for 6, 12 and 24 h at 37°C in an atmosphere containing 5% CO_2_ and 95% air. Following completion of the treatment, cells were washed with phosphate-buffered saline (PBS; Beyotime Institute of Biotechnology) and 50 *µ*l MTT (5 mg/ml; Sigma-Aldrich) was added. The cells were subsequently incubated for 4 h at 37°C to allow for the formation of formazan precipitate, which was dissolved in dimethyl sulfoxide (Sigma-Aldrich). The absorbance at 490 nm in each well was then measured with a Multiskan Ascent ELISA reader (Thermo Fisher Scientific, Waltham, MA, USA). The cell viability was defined relative to that of the untreated control cells as follows: Cell viability = absorbance of treated sample/absorbance of control.

The results of the aforementioned experiments revealed that the optimal concentrations of EGCG and Zn^2+^ were 10 *µ*M and 5 *µ*M, respectively. Subsequently, these optimal concentrations of EGCG and Zn^2+^ were added to three groups of H9c2 cells (group I, 10 *µ*M EGCG; group II, 5 *µ*M Zn^2+^ and group III, 10 *µ*M EGCG + 5 *µ*M Zn^2+^) in order to investigate their toxicity. The treated cells were incubated for 24 h at 37°C and their viability was determined by MTT assays.

### Morphological analysis of H9c2 cells adhering to the plate

H9c2 cells were equally seeded at a density of 2×10^5^ cells/well in six-well plates (20 × 20 mm) for 24 h and were subsequently randomly divided into five groups (A, B, C, D and E). Group A (H/R group) was exposed to 3-h simulated anoxia followed by 1-h re-oxygenation. In group B, EGCG was added at a dose of 10 *µ*M prior to 3-h simulated anoxia followed by 1-h reoxygenation. In group C, Zn^2+^ was added at a dose of 5 *µ*M prior to hypoxia treatment. In group D, EGCG was added at a dose of 10 *µ*M with 5 *µ*M Zn^2+^ prior to hypoxia treatment. In group E, the PI3K inhibitor LY294002 [0.5 *µ*M/0.57 *µ*M/0.97 *µ*M (PI3Kα/δ/β) Sigma-Aldrich] was added with EGCG and Zn^2+^ simultaneously. Cells without treatment were considered as the controls. Subsequent to treatment, microscopic images of H9c2 cells adhering to the plate were captured using an inverted microscope at a magnification of x200 (CKX41SF; Olympus Corp., Tokyo, Japan).

### Hoechst 33258 staining

Cells of groups A–E were washed twice in cold PBS and fixed in 4% formaldehyde (Sangon Biotech Co., Ltd.) at 4°C for 10 min. Subsequently, the fixed cells were washed and labeled with Hoechst 33258 (5 *µ*g/ml) at room temperature in the dark for 10 min. The cells were then observed and imaged using a fluorescence inverted microscope (Eclipse TE2000-S; Nikon Corp., Tokyo, Japan) with excitation at 350 nm and emission at 460 nm.

### Western blot analysis of protein expression

H9c2 cells were seeded in 100-mm cell culture dishes at a density of 2×10^6^ cells/well and incubated according to the aforementioned time-temperature protocol. Cells were lysed in a whole cell extract buffer (Sigma-Aldrich). Protein concentration was determined using a Bicinchoninic Acid Protein Assay kit, according to the manufacturer’s instructions (Beyotime Institute of Biotechnology). Protein samples of the whole cell lysate were mixed with an equal volume of 5X SDS sample buffer (Beyotime Institute of Biotechnology), boiled for 5 min and then separated by 8–15% SDS-PAGE (Beyotime Institute of Biotechnology). Following electrophoresis (Mini-PROTEAN 3 cell; Bio-Rad Laboratories, Inc., Hercules, CA, USA), the proteins were transferred to nitrocellulose membranes (Beyotime Institute of Biotechnology). The membranes were blocked in 5% non-fat dried milk for 1 h, rinsed and incubated with the following antibodies: Mouse monoclonal anti-p-Akt (cat. no. 2920) and rabbit monoclonal cleaved caspase-3 (cat. no. 9664) in PBS containing 0.1% Tween-20 (PBS-T) overnight at 4°C. The primary antibodies were removed by washing the membranes three times in PBS-T, which were then incubated for 1 h with horseradish peroxidase-conjugated goat anti-rabbit (cat. no. 7074) and horse anti-mouse (cat. no. 7076) immunoglobulin G secondary antibodies (Cell Signaling Technology, Inc., Danvers, MA, USA). Following three washes with PBS-T, immunopositive bands were visualized using chemiluminescent reagent (Beyotime Institute of Biotechnology) and exposed to X-ray film (Thermo Fisher Scientific).

### Statistical analysis

Data were expressed as the mean ± standard deviation. Statistical differences were analyzed using a one-way analysis of variance followed by the least significant difference test, to determine significant differences within groups. P<0.05 was considered to indicate a statistically significant difference in all calculations. Statistical analyses were performed using SPSS, version 17.0 (SPSS, Inc., Chicago, IL, USA).

## Results

### Viability of H9c2 cells in the presence of EGCG and Zn^2+^

**Figure 1:**
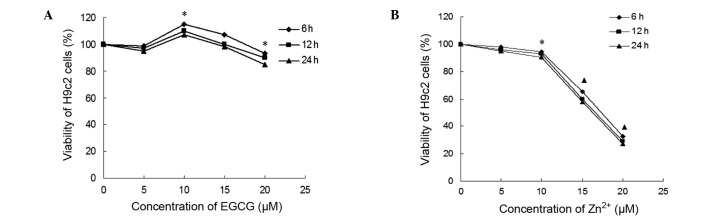
Cytotoxicity of EGCG and Zn^2+^ on H9c2 cells determined by MTT assay. Viability of H9c2 cells following (A) treatment with various concentrations of EGCG (0–20 *µ*M) and (B) treatment with various concentrations of Zn^2+^ (0–20 *µ*M). Data are expressed as the mean ± standard deviation of a minimum of five experiments. ^*^P<0.05 vs. control group; ^▲^P<0.01 vs. control group. EGCG, epigallocatechin-3-gallate.

To analyze cell survival, cells were cultured with various concentrations of EGCG and Zn^2+^. As presented in Fig. 1, treatment with low concentrations of EGCG (<5 *µ*M) and Zn^2+^ (<10 *µ*M) resulted in no significant alterations in H9c2 cell growth. Treatment with 10 *µ*M EGCG significantly increased the viability of H9c2 cells compared with that of the control group. By contrast, treatment with high concentrations of EGCG (>20 *µ*M) resulted in a significant loss of cell viability. Zn^2+^ administration at concentrations of 0–5 *µ*M resulted in no significant alterations in H9c2 cell growth. However, treatment with high concentrations (>10 *µ*M) of Zn^2+^ resulted in a significant loss of cell viability, with a sharp reduction at concentrations of 15–20 *µ*M. Therefore, the optimal concentrations were proposed to be 10 *µ*M for EGCG and 5 *µ*M for Zn^2+^.

**Figure 2:**
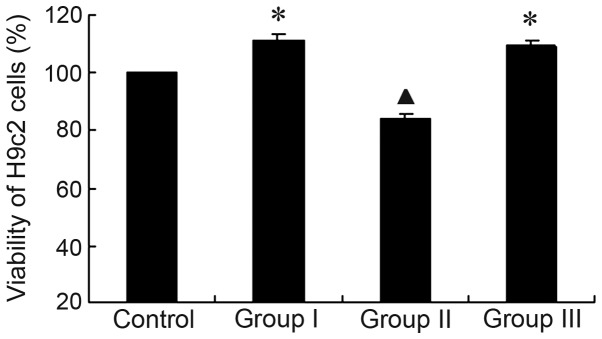
Cell viability of H9c2 cells treated with optimal concentrations of EGCG and Zn^2+^. Group I, 10 *µ*M EGCG; group II, 5 *µ*M Zn^2+^ and group III, 10 *µ*M EGCG + 5 *µ*M Zn^2+^. Values are expressed as the mean ± standard deviation. ^*^P<0.05 vs. control group; ^▲^P<0.05 vs. group I or group III. EGCG, epigallocatechin-3-gallate.

Subsequently, the identified optimal concentrations of EGCG (10 *µ*M) and Zn^2+^ (5 *µ*M) were added to three groups of H9c2 cells (group I, 10 *µ*M EGCG; group II, 5 *µ*M Zn^2+^; and group III, 10 *µ*M EGCG + 5 *µ*M Zn^2+^) in order to investigate their toxicity. As demonstrated in Fig. 2, no significant alterations in toxicity were observed in group III when compared with group I.

### EGCG + Zn^2+^ treatment prevents morphological variations of H9c2 cells following H/R injury

**Figure 3:**
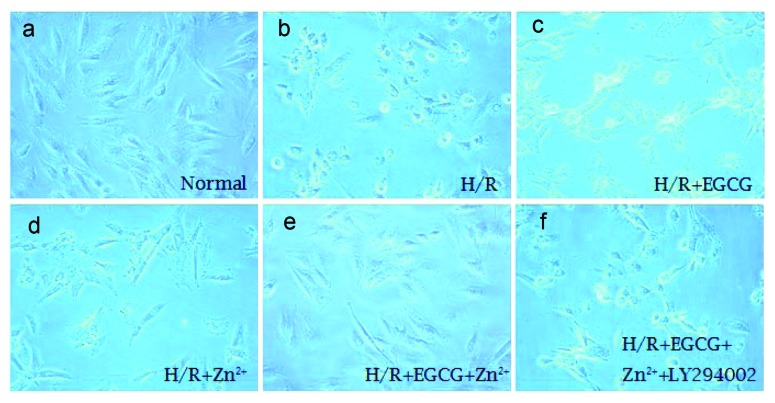
Microscopic images of H9c2 cells subsequent to exposure to 3-h simulated anoxia followed by 1-h reoxygenation (H/R); (magnification, x200). (a) Normal H9c2 cells; (b) group A (H/R), H9c2 cells exposed to 3-h simulated anoxia followed by 1 h reoxygenation; (c) group B, H/R + 10 *µ*M EGCG; (d) group C, H/R + 5 *µ*M Zn^2+^; (e) group D, H/R + 10 *µ*M EGCG and 5 *µ*M Zn^2+^; (f) group E, H/R + 10 *µ*M EGCG, 5 *µ*M Zn^2+^ and the phosphatidylinositol-3-ki-nase inhibitor LY294002. H/R, hypoxia/reoxygenation; EGCG, epigallocatechin-3-gallate.

Morphological variations of H9c2 cells treated with EGCG, Zn^2+^ and EGCG + Zn^2+^ with and without LY294002 were observed through an inverted microscope. As indicated in Fig. 3, normal H9c2 cells adhered to the plate uniformly, with a filamentous shape (Fig. 3a). When exposed to H/R injury, H9c2 cells appeared to exhibit morphological variations, becoming round or irregular in shape, which indicated the cytotoxic capacity of H/R injury (Fig. 3b). Following pretreatment with Zn^2+^, the extent of cell damage was attenuated (Fig. 3d). When exposed to the mixture of EGCG and Zn^2+^, H9c2 cells exhibited no significant morphological variations, as compared with the other groups, with the majority of cells remaining a normal shape following H/R injury (Fig. 3e). No significant differences were observed in cell morphology between the H/R group and the cells treated with EGCG alone (Fig. 3c) or the PI3K inhibitor LY294002 (Fig. 3f).

**Figure 4:**
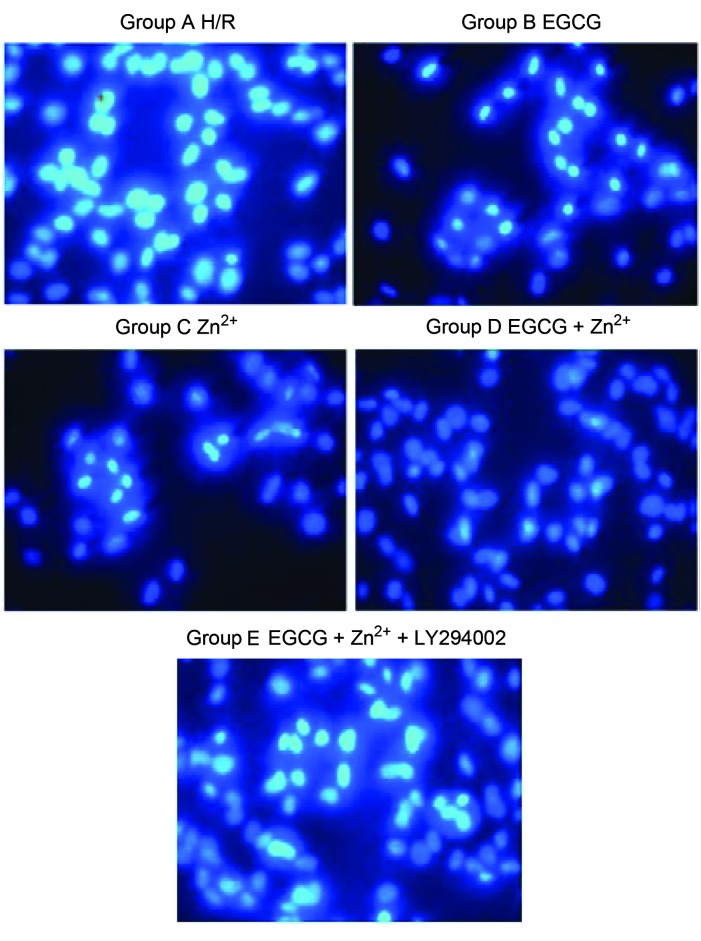
Fluorescent images of Hoechst 33258-stained H9c2 cells following H/R injury in the absence or presence of EGCG and Zn^2+^ in groups A–E. Bright sections indicate the aggregation and fragmentation of chromatin, and shrunken and irregularly shaped nuclei indicate apoptotic cells. Nuclei were stained with Hoechst 33258 (magnification, x100). (A) The H/R group; (B) EGCG (10 *µ*M) group; (C) Zn^2+^ (5 *µ*M) group; (D) EGCG (10 *µ*M) + Zn^2+^ (5 *µ*M) group; (E) EGCG (10 *µ*M) + Zn^2+^ (5 *µ*M) + LY294002 group. H/R, hypoxia/reoxygenation; EGCG, epigallocatechin-3-gallate.

**Figure 5:**
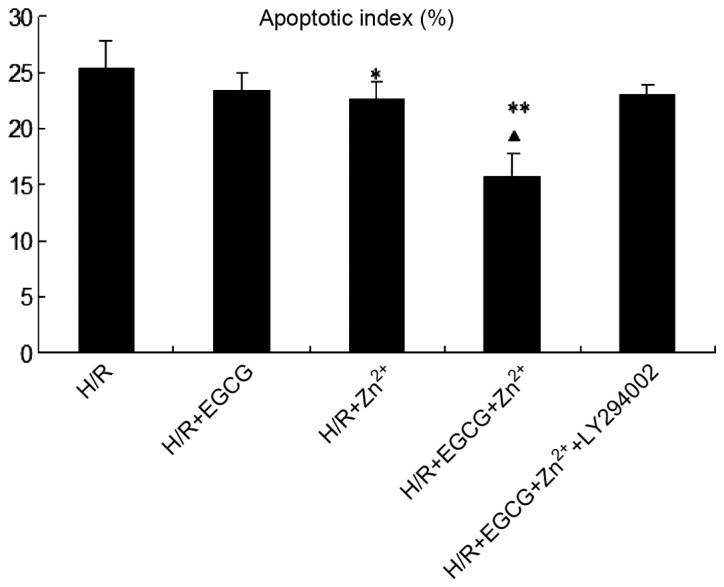
Level of apoptosis following H/R injury and treatment with EGCG and/or Zn^2+^ as determined by Hoechst 33258 staining. Apoptotic index of H9c2 cells was defined by the percentage of apoptotic cells over the total number of cells counted. Values are expressed as the mean ± standard deviation. ^*^P<0.05 vs. H/R group; ^**^P<0.01 vs. H/R group; ^▲^P<0.01 vs. H/R + Zn^2+^ group. EGCG, epigallocatechin-3-gallate; H/R, hypoxia/reoxygenation.

The Hoechst 33258 dye was able to diffuse through the intact membranes of H9c2 cells and stain their DNA, thus assessing the levels of apoptotic cell death. Apoptosis was confirmed by observing specific morphological alterations, including reduction in cell volume and nuclear chromatin condensation. Subsequent to staining, the H/R group was uniformly stained with a significant fluorescent signal, and demonstrated clear apoptotic morphology since the cells were condensed and contained fragmented chromatin in their nuclei (Fig. 4). To investigate whether EGCG and Zn^2+^ were cardio-protective, the number of apoptotic cells was determined and expressed as a percentage of the apoptotic index. The apoptotic index of H9c2 cells was determined as the percentage of apoptotic cells over the total number of cells counted, and a minimum of 500 cells were counted in each experiment (Fig. 5). Treatment with EGCG + Zn^2+^ significantly reduced the apototic index compared with that of the H/R group (P<0.01) and the H/R + Zn^2+^ group (P<0.01).

### EGCG + Zn^2+^ treatment significantly reduces cleaved caspase-3 expression and enhances p-Akt expression in H9c2 cells

**Figure 6:**
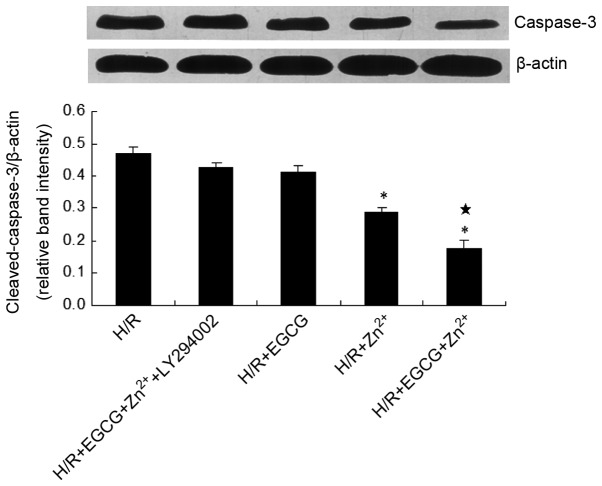
Effects of EGCG and Zn^2+^ on cleaved caspase-3 protein expression levels in H9c2 cells exposed to H/R injury. The expression levels of caspase-3 were determined by western blotting and the ratio of caspase-3/β-actin is presented. H/R, H/R only; H/R + EGCG, H/R with 10 *µ*M EGCG; H/R + Zn^2+^, H/R with 5 *µ*M Zn^2+^; H/R + EGCG + Zn^2+^, H/R with 10 *µ*M EGCG and 5 *µ*M Zn^2+^; H/R + EGCG + Zn^2+^ + LY294002, H/R with 10 *µ*M EGCG, 5 *µ*M Zn^2+^ and the phosphatidylinositol-3-kinase inhibitor LY294002. Results are representative of three independent experiments. Values are expressed as the mean ± standard deviation. ^*^P<0.01 vs. H/R group. ^*^P<0.01 vs. H/R + Zn^2+^ group. EGCG, epigallocatechin-3-gallate; H/R, hypoxia/reoxygenation.

To explore the potential signaling pathways contributing to the anti-apoptotic functions of EGCG and Zn^2+^, the expression of cleaved caspase-3 was examined. As indicated in Fig. 6, treatment of H9c2 cells with Zn^2+^ in the presence or absence of EGCG reduced the expression levels of cleaved caspase-3 when compared with those of the H/R group. Furthermore, the EGCG + Zn^2+^ group reduced the enhancement in the expression levels of active caspase-3 following simulated H/R, compared with those following Zn^2+^ treatment alone. By contrast, with the addition of the PI3K inhibitor LY294002, the EGCG + Zn^2+^ group exhibited no significant differences in caspase-3 expression levels compared with those of the H/R group. Thus, the anti-apoptotic effects of EGCG + Zn^2+^ may occur as a result of reduced processing and activation of the downstream effector caspase-3 in H9c2 cells exposed to H/R, thus leading to a cardioprotective effect.

**Figure 7:**
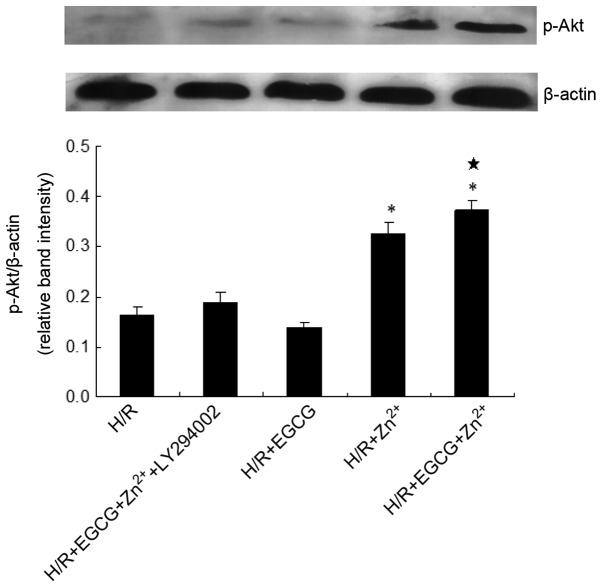
Effects of EGCG and Zn^2+^ on p-Akt protein expression levels in H9c2 cells exposed to H/R injury. The expression levels of p-Akt were determined by western blotting. The ratio of p-Akt/β-actin is presented. Results are representative of three independent experiments. Values are expressed as the mean ± standard deviation. ^*^P<0.01 vs. H/R group; ^*^P<0.05 vs. H/R + Zn^2+^ group. EGCG, epigallocatechin-3-gallate; p-, phosphorylated; H/R, hypoxia/reoxygenation.

Protein expression levels of of p-Akt were measured in order to determine whether EGCG and Zn^2+^ were able to inhibit apoptosis by regulation of the expression of p-Akt via the PI3K/Akt pathway. The results indicated that treatment with 10 *µ*M EGCG + 5 *µ*M Zn^2+^ significantly increased the expression levels of p-Akt protein compared with those of the H/R group. When treated with Zn^2+^ alone, the expression levels of p-Akt were also increased (Fig. 7). The efficacy of the PI3K inhibitor LY294002 was confirmed through the observed reduction in p-Akt. When H9c2 cells were pre-incubated with this inhibitor, the expression levels of p-Akt were significantly reduced compared with those in the H/R + EGCG + Zn^2+^ group (P<0.05). Therefore, these data suggested that the apoptotic effects of EGCG + Zn^2+^ on hypoxic stress-induced apoptosis of cardiomyocytes were mediated, at least in part, through PI3K/Akt signaling.

## Discussion

I/R induces multiple modes of cellular injury and death, and necrosis and apoptosis are suggested to be key mediators. Necrosis describes pathological death of cells resulting in irreversible damage, whereas apoptosis is characterized by ATP-dependent programmed cell death, progressing via signaling pathways that offer potential targets for therapeutic intervention (8). Previous studies have demonstrated that inhibiting caspases limits myocardial injury and specifically individually inhibiting caspase-3, -8 and -9 limits the infarct size in animal models (23,24). Therefore, agents that possess anti-apoptotic activities may provide therapeutic potential for the attenuation of I/R injury. The PI3K/Akt pathway is known to be a target of I/R injury and serves a critical role in cell survival by regulating caspase-mediated apoptosis. In addition, activation of this signaling pathway has been reported to have anti-apoptotic effects in cardiomyocytes and other cell types (25,26).

A previous study demonstrated that the consumption of tea, particularly green tea, is beneficial for the prevention of cardiovascular disease (27). Previous studies have attempted to take advantage of and enhance the desirable bioactivities of green tea (9,28,29). Investigations into the pharmaceutical activities of metal-flavonoid complexes have additionally attracted research interest. The desirable effects of flavonoids, including anticancer and antioxidant effects, have been demonstrated to be enhanced by metals. Kagaya *et al* (30) observed that zinc was able to enhance the hepatoprotective effects of EGCG in isolated rat hepatocytes, and it has also been reported that exogenous zinc protects cardiomyocytes against H/R-induced apoptosis by targeting the PI3k/Akt pathway (18). In addition, previous studies have observed that the pretreatment with EGCG had protective effect on INS-1 cells against oxidative stress via the enhancement of anti-apoptosis signaling through increased levels of phosphorylated PI3K and Akt (31). The effects of EGCG on PI3K/Akt signaling in myoctyes remain to be fully elucidated, and little is known about the actions of EGCG + Zn^2+^ on H/R-induced H9c2 cell growth and apoptosis and the associated intracellular signaling pathways.

The results of the present study indicated that pretreatment with 10 *µ*M EGCG resulted in no significant effects on apoptosis when compared with the H/R group; however, Zn^2+^ treatment reduced the apoptotic index at concentrations of 5 *µ*M. In addition, the apoptotic index following treatment with EGCG + Zn^2+^ was observed to be significantly reduced compared with that following Zn^2+^ treatment alone. The signaling pathways involved in this cardioprotective effect were subsequently evaluated. Furthermore, no significant alterations in the expression levels of caspase-3 and p-Akt were identified in H9c2 cells with EGCG-treatment alone, which implied that EGCG did not exhibit an anti-apoptotic effect through the PI3K-Akt pathway at a concentration of 10 *µ*M. When treated with Zn^2+^ alone, an anti-apoptotic effect was observed through the activation of the p-Akt protein. It was identified that administration of EGCG + Zn^2+^ significantly reduced the expression levels of cleaved caspase-3 protein and elevated those of p-Akt protein in H9c2 cells compared with those in the H/R and Zn^2+^ treatment groups. An investigation using the PI3K inhibitor LY294002 was also conducted, which was used to verify the effect of the PI3K pathway in myocardial apoptosis. Pretreatment with LY294002 and EGCG + Zn^2+^ did not further reduce caspase-3 activity or increase p-Akt expression following H/R injury.

The results of the current study suggested that the PI3K/Akt pathway may be the major pathway underlying EGCG + Zn^2+^, with regard to the prevention of reperfusion injury and myocardial apoptosis. Notably, zinc supplementation with EGCG effectively enhanced the p-Akt levels following H/R, resulting in increased cell viability. The results suggested that the interactions between EGCG and zinc may preserve and enhance their anti-apoptotic effects via the PI3K/Akt pathway. The prophylactic administration of pharmacological agents mimicking the effects of ischemic preconditioning are suggested to represent an effective method of reducing the extent of myocardial damage resulting from I/R or H/R injury. The current study aimed to evaluate the anti-apoptotic activity of EGCG + Zn^2+^, with the objective of discovering an effective and safe cardioprotective agent for the prevention and/or treatment of I/R injury. Zinc acetate was previously approved by the Food and Drug Association in 1997 as a drug for the treatment of Wilson’s disease, which indicates the clinical safety of zinc supplementation (32). Further understanding of the safety and efficacy of EGCG + Zn^2+^ in clinical practice would be beneficial.

The results of the current study suggest that the inhibition of I/R injury may provide opportunities to improve the function and viability of H9c2 cells, through exhibiting an anti-apoptotic effect. It was demonstrated that when EGCG interacted with zinc, the cardioprotective activity was significantly enhanced, as compared with EGCG treatment alone, potentially via activation of the PI3K/Akt pathway. In conclusion, EGCG and zinc may represent a potent therapeutic agent for I/R injury.
